# Provider perceptions and experiences integrating depression treatment into chronic healthcare services in Neno District, Malawi: a qualitative study

**DOI:** 10.1186/s12913-025-13830-2

**Published:** 2025-12-10

**Authors:** Myrrah Kamwiyo, Owen Mwale, Kondwani Mpinga, Waste Kayira, Ian Johnson, Todd Ruderman, Beatrice Matanje, Karen Kasambara, Amruta Houde, Rachel Isaacs, Cecilia Needham, Glenn Wagner, Kazione Kulisewa, Michael Udedi, Ryan K. McBain

**Affiliations:** 1 Partners in Health, Neno, Malawi; 2 Union College, Lincoln, NE USA; 3 Partners in Health, Boston, MA USA; 4 Harvard Medical School, 20 Park Plz, Boston, MA 02116 USA; 5 RAND, Santa Monica, CA USA; 6 Kamuzu University of Health Sciences, Blantyre, Malawi; 7 Ministry of Health, Lilongwe, Malawi; 8 Healthcare Delivery, RAND, Washington, DC USA; 9 Tufts University School of Medicine, Boston, United States

**Keywords:** Antidepressant therapy, Problem management plus, Non-communicable diseases, Integrated chronic care clinics

## Abstract

**Background:**

In low-resource settings, integrating mental health services into existing care platforms is critical to expanding access and improving quality of care. In Neno District, Malawi, the Integrated Chronic Care Clinic–Depression module (IC3D) clinical trial introduced depression screening and treatment, including antidepressant therapy (ADT) and group Problem Management Plus (PM+), within chronic care services. This study explored healthcare and lay providers’ experiences and perceptions of integrating depression care for patients with chronic conditions at these clinics.

**Methods:**

We conducted an exploratory qualitative study using semi-structured key informant interviews with 18 depression care providers (eight clinical officers and ten counselors). Interviews were transcribed, translated from Chichewa to English, and analyzed thematically using Dedoose 9.0 to identify patterns in these providers’ perceptions of effectiveness, facilitators, barriers, and recommendations for care integration.

**Results:**

Four key themes emerged from the interviews. First, providers reported positive perceptions of intervention effectiveness, noting improvements in patients’ mental, physical, and functional health, including blood pressure control and HIV viral suppression. Second, providers shared constructive feedback on elements of intervention facilitation, including the PM + user guide, group format, and ongoing training and mentorship. Third, providers identified key challenges which included transportation barriers, increased workload, and missed appointments. Finally, providers recommended ways to improve care delivery, such as allocating additional resources as well as expanding infrastructure and depression screening at additional patient entry points such as outpatient departments and palliative care.

**Conclusions:**

Based on provider testimony, the integration of depression care into chronic care services in Neno District, Malawi is perceived as feasible, acceptable, and impactful. Their insights underscore the value of structured mentorship, task-shifting, and holistic care approaches in low-resource settings. These findings will be used to inform government-led district-wide strategies and policy for strengthening behavioral and pharmacologic treatment of depression in Neno District, including recommendations to address logistical constraints, ensure consistent supervision and documentation, and expand screening access. Future research should explore strategies to improve the integrated depression model, including accounting for comorbid mental health conditions, and expanding access to screening and care.

**Trial registration:**

The trial was registered with Clinical Trials. Gov: NCT04777006 (Registration Date: February 23, 2021).

**Supplementary Information:**

The online version contains supplementary material available at 10.1186/s12913-025-13830-2.

## Background

The World Health Organization (WHO) advocates for the integration of healthcare systems to improve access to care and to promote health outcomes [1]. Integration has also been shown to improve job satisfaction for healthcare workers as a function of expanding their competencies and diversifying daily tasks [1].

Several countries have recently introduced an integrated care approach to health care services, including merging chronic care [2] and HIV/AIDS services [3]. By comparison, information on the integration of mental health care services is limited in low- and middle-income countries (LMICs). Based on the magnitude of the treatment gap for addressing mental health conditions, which is greater than 75% in many countries [4], service integration has the potential to generate an impact on access to mental health services. One study in South Africa [5] worked on developing a district mental health care plan by integrating depression among chronic illnesses and developed collaborative care packages to enable integration at the facility and community levels. However, the authors indicated a need to leverage human resources and implementation tools to better facilitate such integration practices.

Although some countries in sub-Saharan Africa are working to integrate mental health care into chronic care, there are several challenges that exist in service delivery. In South Africa, despite integrating mental health care into primary health care settings (PHCs) in KwaZulu-Natal Prince, implementers faced several challenges, including a lack of training in mental health care among staff working in PHCs, unavailability of mental health policies, inadequate resources, and poor communication among management [6].

Alongside service integration, task shifting has been promoted by experts as a solution to close the treatment gap, with non-specialists being trained to offer select mental health interventions [7]. To promote success, non-specialists often administer standard screening instruments under the supervision of and referral to specialists for diagnostic confirmation [8]. Task-sharing of mental health services is perceived to be acceptable and feasible in LMICs as long as key conditions are met, such as increased human resource capacity and improved access to medication [9].

Among common mental health conditions, major depressive disorder (MDD) is a paramount concern, representing a leading cause of disability in much of sub-Saharan Africa [10]. In Malawi, past evidence suggests the prevalence of MDD is 18% among individuals with chronic health conditions [11]. However, the treatment gap remains high [12], largely due to a dearth of trained mental health care providers. This disparity underscores the need for the public sector to capacitate existing cadres of healthcare and lay providers to offer mental health services through an integrated approach. Positive impacts of depression care integration have been documented in other settings and range from positive impacts on the overall quality of care delivery to improved self-care by patients [7].

Many contextual factors have been identified as potential determinants of providers’ capacity to provide high-quality care during service integration [13] such as additional workload [14], training support [15], job satisfaction, and compensation [16]. Such factors, when overlooked, can have a negative impact on the quality of care and worker motivation. Providers are positioned to comment about whether their needs are being met, share their perceptions on the feasibility and acceptability of integration, and provide guidance on how to strengthen service delivery [17].

This study aims to provide insights on the perceptions and experiences of Problem Management Plus (PM+) providers, offering an opportunity to learn from their unique feedback, including challenges and suggestions. The study is couched within a broader clinical trial in a rural district of Malawi, which aims to assess the effectiveness of a stepped-approach to MDD treatment that comprises group PM + and antidepressant therapy [18]. Additionally, it complements insights offered from another study that focuses on the feasibility and acceptability of PM + from the patient perspective [19] as well as the study on the randomized controlled trials cost analysis of integrating depression treatment [20]. We hypothesized that, as a result of adaptations to the local setting, group-based PM + and antidepressant therapy (ADT) would be deemed appropriate by providers for broader-scale implementation, and that they would offer unique perspectives and suggestions for strengthening this model of care.

## Methods

### Study setting

Neno District is one of the most remote districts in the country of Malawi, due to its distance from major cities and poor road infrastructure. Partners in Health/Abwenzi Pa Za Umoyo, in partnership with Malawi’s Ministry of Health, started operating in Neno District in 2007 and launched Integrated Chronic Care Clinics (IC3) in 2014, with the goal of integrating screening and treatment for non-communicable diseases into Malawi’s HIV platform. Neno District has 14 health facilities providing IC3 biweekly. According to data from the Neno District Electronic Medical Records (EMR), the Integrated Chronic Care Clinic (IC3) serves a population of over 14,000 active patients. The clinic is staffed by a core team comprising nurses and two permanent clinicians, each responsible for managing their own clinic. To ensure continuity of care during their absence, six additional clinicians are available from other sections to provide support. On average, a single clinician attends to more than 100 patients per clinic. A cadre of clinicians was introduced in Malawi following the critical shortage of physicians below the WHO recommended threshold of 0.049 per 1000 which despite the introduction of the clinical officers, the workload still remains high [21–23].

In 2021, the randomized controlled trial corresponding to this study introduced MDD screening and treatment to the IC3 clinical model. Providers conducted depression screening during routine patient screenings at IC3 for adults diagnosed and treated with chronic medical conditions such as HIV, diabetes, hypertension, asthma, and epilepsy using the Patient Health Questionnaire (PHQ-9 and PHQ-2), a validated screening tool for assessing depressive symptoms in Malawi. The PHQ-9 was administered electronically using tablets provided to psychosocial counselors, allowing direct data entry into CommCare. Patients with moderate or severe symptoms (PHQ-9 scores ≥ 10) who met DSM-IV criteria for current MDD were enrolled in the clinical trial, with their treatment plan determined by their symptoms. Those with moderate depression symptoms were recommended group PM + alone and those with severe symptoms were recommended a combination of group PM + and ADT. The PM + intervention, designed for mild and moderate depression, was conducted in groups of 2–8 participants and comprised five weekly sessions, each lasting approximately 90 min. Trained psychosocial counselors were responsible for group organization, patient communication, and conducting the sessions. Sessions were conducted at locations convenient for patients, with groups organized based on geographical proximity. Psychosocial counselors included both counselors with bachelor’s level degrees, as well as members of the local community with no prior experience (lay counselors). Patients were not told which counselor corresponded with which background. All counselors participated in a week-long core skills training on the PM + intervention and human subjects research, and participated in refresher trainings on a quarterly basis. Mental health clinicians provided random clinical supervision, including completion of a PM + fidelity checklist, with each counselor. Those who were enrolled in ADT additionally were referred to clinical officers at the nearest health facility. Clinical officers prescribed fluoxetine, ensured patients had an adequate medication supply, monitored side effects, and conducted follow-up visits to assess the need for dosage titration.

In this study, researchers investigated providers’ experiences delivering PM + and ADT during the clinical trial. We interviewed eight clinicians and ten psychosocial counselors. The clinicians were based at two secondary care facilities: Neno District Hospital and Lisungwi Community Hospital. In addition to providing care at IC3 clinics at these hospitals, the clinicians also traveled biweekly to conduct outreach clinics at 12 primary care health centers. The most distant clinic is located 34 km from the main hospital, and providers traveled along poorly constructed roads to deliver care to patients in these remote areas.

### Study design and sample

This study used an exploratory qualitative design with purposive sampling, enabling providers to share their experiences providing depression care through in-depth interviews. Interviews were semi-structured, including a protocol that inquired about provider experiences and perceptions according to three domains: (i) perceptions of MDD as a medical condition, (ii) facilitators and challenges to executing care integration of MDD services, and (iii) any feedback they wished to provide on the model (see the Online Supplement for the full protocol). Two cadres of providers were approached for interviews: clinical officers and psychosocial counselors (see Table 1).

**Table 1:** Training & educational levels of study participants

Cadre of providers	Range of work experience	Level of education	Roles and responsibilities
Lay psychosocial counselors	3–4 years	Secondary/High School Education - Malawi School Certificate of Education (MSCE)	• Screening for depression using the PHQ-9 screening tool • Facilitation of group PM + sessions
Professional psychosocial counselors	5–7 years	Tertiary Level (2-year University Diploma in Psychosocial Counselling)	• Screening for depression using the PHQ-9 screening tool • Facilitation of Group PM + sessions • Conducting one-on-one PM + sessions for special cases • Co-supervising and mentoring lay counselors
Clinical officers	5–15 years	Tertiary Level (3-year University Diploma + 2-year Degree in Clinical Medicine)	• Prescription of ADT • Completing information in patients’ health records, known as Mastercards.

All providers who were approached for interviews completed the interview process. In Malawi, clinical officers were introduced in 1985 to address the severe shortage of medical doctors [19, 20], and they provide the majority of diagnostic and treatment services in the country. Psychosocial counselors are Malawi’s primary cadre of mental health providers for behavioral services. They are equipped to provide patients with psychosocial expertise with cultural understanding, foster tailored interventions and contribute to the mental well-being of Malawians [24].

Despite efforts to improve mental health care in the country, there is still a significant shortage of providers who deliver mental health care [25]. In this study, we interviewed both lay and professional psychosocial counselors who took an active part in depression screening and PM + provision [26]. Providers were invited to participate in interviews based on three criteria: they were 18 + years old; they had participated in the delivery of depression care services at IC3 clinics in Neno district for six or more months over the trial period; and they were open to being interviewed by a research assistant. Providers were informed that their interviews would be anonymized and that audio files and transcripts would be encrypted and stored on a password-protected computer. Those who expressed interest were administered written informed consent.

### Ethical considerations

Before the study commenced, the research team obtained approval from the Neno District Health Research Committee (NDHRC) and the National Health Sciences Research Committee (NHRSRC). The RAND Corporation Human Subjects Protections Committee in the United States also reviewed and approved the study protocol. Study participants granted consent before interviews by signing the informed consent forms. All elements of the study were conducted in accordance with the Declaration of Helsinki.

To promote the integrity of the information in this study, we ensured all participants were provided with adequate information on the study design, contents, risks, and benefits through written informed forms, thus demonstrating voluntary participation without coercion. Identification numbers were used for all participants to ensure confidentiality and interviews were conducted in a private office out of earshot from others. The study participants did not receive money as a form of incentive to take part in this study. The interviewer was selected as an individual outside the clinical trial and supervisory structure of interviewees in order to promote impartiality and confidentiality of disclosed information. This individual was trained in qualitative interviewing techniques and followed a semi-structured interview protocol to ensure consistent coverage of topics across interviews.

### Data collection procedures

Providers were individually approached and invited to participate in key informant interviews. In total, 18 individuals who met eligibility criteria were invited: eight clinical officers and ten counselors. Among the counselors, four were professional counselors with specialized mental health training and six were lay counselors with one week of training on the manualized protocol for group PM+. All agreed to participate.

Following consent procedures, the interviewer and interviewee identified a convenient time and location for the interview. All interviews were held in a private location where the interviewee could speak freely. The conversation was conducted in Chichewa (the local language) or English, depending on the interviewee’s preference, and an audio recorder was used to capture content. Typically, interviews lasted 30 to 60 min.

### Data analysis

Recorded interviews were transcribed verbatim by the research team and, when necessary, forward- and back-translated from Chichewa to English. Transcripts were then uploaded to Dedoose, a qualitative software platform used for analysis. The analytic process followed a standard pattern of open and axial coding. In the open coding phase, researchers systematically examined and categorized data (line-by-line codes) to identify emergent concepts, themes, and patterns. Once an initial set of codes was developed, research team members engaged in axial coding, linking connections between categories, developing subcategories, and refining the coding structure until concluding with a coherent framework for explaining relationships. We aimed to recruit participants until we achieved saturation, defined as the point at which new themes or insights from analysis of additional interviews stopped arising.

## Results

In total, 18 individuals agreed to participate—including eight clinical officers, 5 professional counsellors, and six lay counsellors. Additional demographic characteristics can be found in Table 2.

**Table 2:** Demographic characteristics of study participants

Characteristic	Frequency (%)
**Sex**	
Male	13 (72%)
Female	5 (28%)
**Age**	
21–30	10 (56%)
31–40	6 (33%)
41–50	2 (11%)
**Provider type**	
Clinical officer	8 (44%)
Professional counselor	4 (22%)
Lay counselor	6 (33%)

**Fig. 1:**
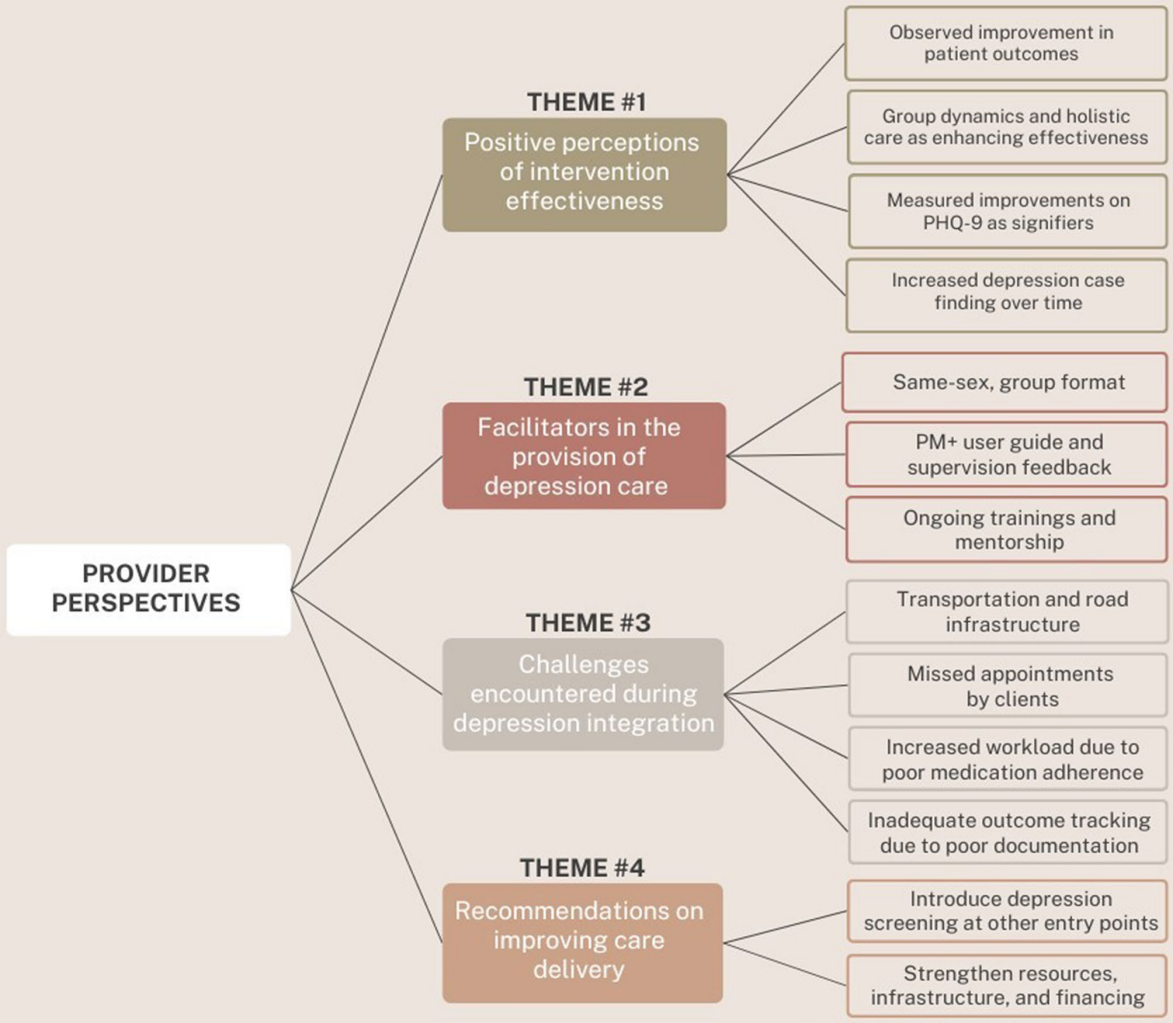
Emergent themes and sub-themes from provider key informant interview

Based on data analysis, four themes emerged from the study: positive perceptions of intervention effectiveness in treating MDD, facilitators in the provision of depression care, challenges encountered during depression integration, and recommendations on improving care delivery. Figure 1 provides an overview of this taxonomy, including the sub-themes that emerged within each of these.

### Theme 1: Positive perceptions of intervention effectiveness

#### Observed improvements in mental health, physical health, and functional outcomes

Providers expressed positive views regarding the effectiveness of depression care for adult patients receiving treatment. At a fundamental level, providers perceived the intervention as effective because they observed clinical improvements in depression symptoms, including symptoms that had not been the patient’s primary concern. For example, one provider made the following remarks: *“Behavioural activation I think it was very helpful because*,* a lot of them had low moods*,* so they couldn’t do things that they used to do in the past*. S*o*,* when we told them the behaviour activation part*,* it helped so much.” (Lay Psychosocial Counselor: No. 5).* In a similar manner, another provider reflected, *“There was a palliative care patient who was screened through the process and found to be depressed. She was further given antidepressant therapy. After that*,* our interaction with her in the clinic changed. I would attribute the improvement to addressing her depression*,* which we had not been looking at critically.” (Clinician: No. 18).* These reflections indicated a perception of MDD as an independent medical condition, rather than a symptom of another illness.

The observations extended beyond mental health symptom improvement and recognition of MDD in patients. Providers highlighted enhancements in patients’ overall physical health which were assessed through disease-specific metrics. For instance, in managing hypertension, blood pressure readings served as biomarkers for physical health progress. Similarly, for individuals receiving Antiretroviral Therapy (ART) for HIV care, changes in viral load measurements were attributed to the integration of depression care in the chronic care clinic. For instance, one provider stated: *“There was a certain client who had thoughts of ending his life*,* but after giving him Problem Management Plus*,* he picked himself up*,* and as of now he is alive and even if you meet him now*,* his health*,* his life*,* is going well. Even when they measure viral load*,* the results come out well now.” (Lay Psychosocial Counselor: No. 12).*

Some aspects of this attribution may also be related to the psychosomatic manifestation of depression, which is widely observed in Malawi. This manifestation occurs when depressive symptoms present in a physical manner, such as trouble sleeping and changes in eating habits. One provider shared; *“Most of the people who had sleeping issues*,* or their sleeping pattern was very bad*,* testified that the intervention helped them sleep” (Lay Psychosocial Counselor: No. 5).* Some providers hypothesized a tentative connection between depression care and improved functional outcomes such as employment status, thus associating socioeconomic outlook and the status of one’s mental health. For example, one provider remarked; *“Most of the patients in the study*,* complained of lack of resources to start up business*,* but throughout the PM + session they were empowered such that some started businesses and others started farming which have been assisting their daily life comparing to the time they were always waiting for well-wishers to provide their daily meals.” (Lay Psychosocial Counselor: No. 12).*

#### Group dynamics and holistic care as contributors to intervention effectiveness 

Two key components of service delivery were believed to enhance the effectiveness of depression care. The first was a recurrent perspective shared among PM + counselors that the group-based format of PM + conferred unique benefits such as opportunities for knowledge exchange and peer support. For example, one provider expressed the following: *“I felt it was a good approach because*,* if there are more than two people*,* they feel encouraged and can learn from their peers’ ideas*,* making it easier for them to tackle their problems compared to working with an individual” (Professional Psychosocial Counselor: No. 14)*.

A second aspect credited with enhancing the effectiveness of service delivery was the comprehensive approach to the services provided. Beyond medical care, patients attending IC3 benefited from additional support. This holistic approach ensures that the medical, emotional, and material needs of the patients are addressed, contributing significantly to the overall effectiveness of depression care. For example, patients who needed urgent financial assistance were provided financial support through Partners in Health (PIH)/Abwenzi Pa Za Umoyo (APZU)’s Program on Social and Economic Rights (POSER). One provider elaborated on this: *“We spent ample time with patients*,* and if needed*,* the counselor would visit their homes. Furthermore*,* if patients faced financial difficulties*,* our team was there to assist them. Thus*,* we established a support system for patients suffering from depression*,* which included medication*,* counseling*,* and financial aid” (Clinician: No. 17).* This sentiment about holistic care for patients was echoed and summarized by other providers; *“This program is good because it looks at the person as a whole*,* not just the medical aspects” (Clinician: No. 16).*

However, not all providers agreed on this topic. One provider shared that the team could improve the level of the social support offered to patients in the program, expressing: *“The way I understand it is that they are focusing on the biological part to patients diagnosed with severe depression by providing antidepressants and to those with psychological issues*,* are provided with psychosocial counseling. Yeah*,* that’s good. But the social part*,* I feel needs more attention…” (Clinician: No. 15).*

#### Measured improvements on PHQ-9 scores as signifiers

Some providers perceived that the integration of depression care was effective because of improvements in clinical outcomes, as measured by the PHQ-9 scale. One provider stated: *“I think it was very effective because a lot of clients that we saw*,* maybe after three months of follow up or six months*,* most of them would come out with… reduced PHQ-9 scores*,* so for me I think it was very helpful*” *(Lay Psychosocial Counselor: No. 5).* Additionally, another provider agreed: *“I noticed changes in patient’s symptoms after PM + when they came to the clinic for a review*,* this was evidenced by the reduced PHQ-9 scores as well as the mental state examination which we assessed as clinicians”* (Clinician: *No. 18).*

#### Increased depression case findings through screening

Before the introduction of integrated care for depression at IC3 in Neno district, there were sparse cases of depression registered at the facilities. However, after providers were trained to screen and manage depression, providers witnessed a stark increase in patients with depression. Of the 15,562 patients at IC3 who were screened for the study, 487 patients screened positive and were invited to enroll in depression care. Thus, the increase in depression cases correlated with increased case detection. One provider shared: *“Through the program*,* we identified new conditions in patients who had been visiting the clinic for a long time. When the study came in*,* we discovered that the same clients that came had depression. The program has been effective. Without the study some people would have committed suicide” (Clinician: No. 17).* Another provider emphasized the importance of increased screening, stating: *“We have managed to dig out depression… We wouldn’t think that they are depressed until they were assessed” (Clinician: No. 6).*

### Theme 2: Facilitators in the provision of depression care

#### Same-sex group format

Providers identified multiple factors they believed enhanced the integration of depression care, including the intervention format. They observed that the group setting made it easier for clients to devise solutions to their problems through the exchange of ideas. Facilitation of these groups was conducted by providers who were of the same sex as the patients, which further fostered an environment where individuals felt comfortable being open and sharing their experiences with peers. For example, one provider reflected: *“A good number of clients were able to participate because it was man to man. They were able to open up sharing their experiences and it went like that. Through my experience*,* it was an added advantage because they were able to express themselves*,* and mind you it’s hard for men to express themselves. But I have observed during PM + they were able to share their experiences…” (Professional Psychosocial Counselor: No. 9).*

#### PM + user guide and supervision feedback

Providers also shared that a written manual and supportive supervision were critical to the intervention’s success. One lay counselor noted the ease of delivering PM + with the aid of a provided manual, which facilitated the management of group sessions: “*As I mentioned earlier*,* I am a lay counselor*,* I have never been in this society. At first*,* when conducting the sessions*,* we faced challenges*,* pressure*,* and some fear. But we were provided with guidebooks” (Lay Psychosocial Counselor: No. 10).* Concerning supportive supervision, a different provider shared: *“The program was effective because supervisors were supportive and accommodating. When we had challenges*,* they were there*,* and they would come in good time. They were available all the time to provide refreshers and help us when needed” (Clinician: No. 17)*. Likewise, a third counselor remarked: *“It wasn’t bad*,* it was okay because the supervisor usually would come when I am seeing the patient*,* so it was nice because whenever I am missing*,* whenever I am not doing better*,* actually the supervisor will say to me: “No*,* I think [in] this scenario*,* you have to do you [it] this way next time you have to do this*,* so it wasn’t bad*,* it was okay.” (Clinician: No. 2).*

#### Routine trainings and mentorship

The APZU mental health team of psychologists and clinicians provided regular mental health training and mentorship to nurses, clinicians, data technicians, and data clerks at IC3. The majority of providers indicated that their ongoing training and mentorship were critical for supporting their capacity to integrate depression care. One provider voiced: *“The project itself nurtured clinicians and nurses to think about depression more often. One aspect that has made people more willing to engage in this process is the package they gained through the trainings provided to us. Now and again there have been series of mentorship as well as regular updates” (Clinician: No. 18).* Others shared that the overall availability and presence of mentorship were positively received. One provider stated: *“The mental health clinical officers were available most of the time*,* if not available physically*,* they were available on the phone if you want to consult them and they were most of the time checking what other clinicians have been doing” (Clinician: No. 6).*

### Theme 3: Challenges encountered during depression integration

Despite the factors that promoted the integration of depression care at IC3 among patients with chronic medical conditions, providers faced constraints that impacted their capacity to deliver the program effectively. While solutions were implemented to address certain challenges and enhance care delivery, others proved more difficult to resolve.

#### Transportation and road infrastructure

Providers faced logistical challenges, exemplified by difficulties in transportation that resulted in their late arrival at PM + venues. Neno District lacks tarmac roads and has a challenging mountainous terrain. One provider lamented: *“Currently I have been using a motorbike but previously we were using vehicles. It was really hard because of limited resources: the same vehicle that was sent to go to a clinic would proceed to PM + places… combined trips meant other staff had to be dropped at the clinic and then would make some delays along the way” (Professional Psychosocial Counselor: No. 3).* Providers shared that, although the transportation challenges were beyond the scope of the care delivered, these challenges impacted patient-provider relationships. One individual shared: *“People could lose trust in us… We could see others would be absent just because we were going there late. All the time*,* we were going there late” (Lay Psychosocial Counselor: No. 11).*

Other providers also shared that transportation shortages and road conditions impacted the delivery of care; however, they stated that the presence of motorbikes improved the situation. One individual shared: *“Sometimes*,* there could be a delay in transport*,* but we learned how to ride the motorbike anyway” (Professional Psychosocial Counselor: No. 14).* Another provider supported this claim: *“To my side*,* I would say transportation was not an issue just like I have stated earlier on that I am a rider*,* a motorbike rider*,* so obviously my errands most of the times were like managed by myself*,* comparing to the time we were using the vehicles” (Professional Psychosocial Counselor: No. 3).*

#### Missed appointments by clients

Patients attending IC3 received appointment dates based on their condition’s progress. For instance, those on ART receive a six-month supply of medication when their viral load results are favorable. As part of the depression integration, clinicians reviewed clinical trial participants’ cases every three months during patient return visits and re-administered the PHQ-9 screening tool to monitor depression symptoms. This follow-up was provided post-PM + intervention and was integrated into routine IC3 clinical activities, aligning with follow-up visits for other chronic conditions, such as HIV medication management. Consequently, patients who received a six-month medication supply may have perceived less immediate need to attend their three-month return interviews, leading to an increase in missed appointments. To address this issue and enhance patient care, counselors conduct home visits using motorbikes for client assessments. One of the providers elaborated: *“Follow-up? I think it had some issues… You find that you may conduct the interview and explain well to the client that they’re supposed to come after three months. But maybe you find that the date that you have just given them is contrary different from the date that this client will come… When it comes to ART*,* they were given maybe six months.” (Professional Psychosocial Counselor: No. 9).* Home visits were essential to integrating mental health care, as providers could follow up with patients who missed their review dates. However, providers encountered challenges in providing such outreach because many patients reside in remote areas, as one provider stated: *“Challenges during home visits included clients living in remote areas without cell phones… We would schedule to meet them on certain days and when we get there would not meet them” (Professional Psychosocial Counselor: No. 14).*

#### Increased workload due to poor medication adherence

Providers at IC3 shared that integrating depression meant that there was additional work beyond their typical duties. Some providers mentioned that the increased workload resulted in poor documentation, which occasionally led to incomplete treatment regimens and some patients in the clinical trial defaulting on their depression medication. One individual reflected: “*It was happening that a client should start ADT*,* but due to work overload*,* you haven’t noted that one needs ADT” (Clinician: No. 15).* However, most providers shared that, despite the additional work, they were able to adapt. For instance, one provider shared: *“At the beginning*,* I faced challenges adapting to the new medication process*,* as it was an additional workload. Initially*,* it was exhausting. But over time*,* we adapted*,* and everything proceeded smoothly*,* just like with other responsibilities” (Clinician: No. 17).*

#### Inadequate outcome tracking due to poor documentation

Another challenge that arose related to data collection and documentation. Clinicians in the IC3 clinic system utilize Mastercards to facilitate the process of medication prescription. The Mastercards serve as documentation tools where clinicians record essential patient information. Providers shared that they encountered shortages of mental health Mastercards in some outreach clinics across Neno. One provider shared: *“Sometimes we do run out of Mastercards and it’s difficult maybe for the EMR guys to capture the data… so maybe making sure that there are no breaks in the availability of Mastercards?” (Clinician: No. 4).*

The APZU mental health team conducted ongoing refresher trainings for providers to address data collection challenges, which improved the situation but did not entirely rectify the Mastercard-related errors. Another provider added: *“I think the other challenge was from the prescribers*,* it was difficult to capture data. People were not filling in the Mastercards properly. Yeah*,* but I think we had some talks*,* they invited the clinicians*,* we had a refresher and everything and kept reminding them about the entry of the Mastercard’s…” (Clinician: No. 4).*

### Theme 4: Recommendations on improving care delivery

Based on their experiences integrating depression care, the providers formulated several recommendations aimed at enhancing operations of the program. After reflecting on both the value of care and challenges in delivery, they proposed the following suggestions:

#### Introducing depression screening at other entry points

Depression screening has been introduced at IC3; however, some of the participants suggested that there are additional opportunities for mental health care screening and integration, including the outpatient department (OPD), palliative care, the Paediatric Development Centre (PDC), and the advanced non-communicable disease clinic. For example, one of the providers explained: *“Currently*,* the OPD focuses on issues like COVID*,* hypertension*,* diabetes*,* malnutrition*,* and TB. Integrating depression screening could help identify patients who may not be physically ill but are experiencing psychosocial issues or depression” (Clinician: No. 8).*

#### Strengthening resources, infrastructure, and financing

Based on the challenges that providers experienced, they suggested improvements around human and monetary resources, such as increasing the number of personnel providing care and having additional vehicles for patient follow-up. Providers also emphasized the importance of having a discrete space for PM + sessions. One provider recommended the following: *“The other thing I would advise them*,* I think*,* is to look into resources. There’s a need for finances*,* human resources*,* as well as infrastructure-wise*,* so the finance can cover for the human resources as well as the medications*,* and the stationary.” (Clinician: No. 4)*.

Another individual added a note about secure spaces and confidentiality, stating; *“Set up… if it is possible*,* maybe a nice location can be found*,* like the way we have sat here right*,* a free place*,* not that we should be in a place of noise in which when discussing with clients*,* they start listening to other stuff surrounding them? A place we can sit as a group and do the needful*,* not one that is maybe too open that those passing by can hear what you are discussing” (Professional Psychosocial Counselor No. 7).*

## Discussion

In this study, psychosocial counselors and clinical officers at IC3 shared experiences and perceptions regarding the integration of depression care into ongoing chronic care services. Although the cadres of providers played different roles in patient screening, treatment, and management, they shared the unified goal of striving to improve the mental and physical health of their patients. Overall, we found that providers articulated positive perceptions regarding the integration of depression care, despite it being a new initiative. They acknowledged the impact on health outcomes, detailed methods that facilitated their work, shared challenges to delivering services, and provided insightful recommendations.

When reflecting on the effectiveness of depression integration, providers pointed to evidence of improvements in patients’ mental, physical, and functional outcomes, including blood pressure for hypertension and viral load for HIV. Research has shown that integrating mental health services, including depression care, into primary care settings can lead to enhanced physical and mental health outcomes [27]. Depression often coexists with other medical conditions; thus, addressing it has the potential to positively impact the management of those comorbidities. For example, a study by Martin and colleagues (2005) found that patients with chronic medical conditions (including depression) that received integrated care had better adherence to medical treatment regimens and improved health outcomes compared to those receiving usual care [28]. Likewise, a meta-analysis by Grote and colleagues (2010) summarized substantial evidence that treatment of depression can lead to improvements not only in mental health but also in physical and functional outcomes [29]. Providers’ perceptions in this study aligned with previous literature indicating that mental health integration improves overall health outcomes.

According to providers, the dynamics within group sessions were critical in the success of PM+. Prior research has shown that group settings enable participants to exchange ideas and draw insights from the experiences and coping strategies of others [30]. Providers reinforced this notion, indicating that patients reported a high level of satisfaction with the group counseling approach. They highlighted the motivational aspect of hearing others’ stories and the learning opportunity the group provided for managing diverse challenges. Similarly, providers shared that same-sex participation and facilitation supported positive outcomes. A study by Malla and colleagues (2019) likewise reported that groups with same-sex participants aided the success of treatment, though the intervention was set in Kashmir [31]. Lastly, providers were also able to monitor and observe the success of treatment quantitatively through reduced PHQ-9 scores. They felt the tool was simple, rapid, effective, and reliable, consistent with evidence from past studies [32]. This feedback also allowed counselors to determine whether one-on-one counseling or other interventions may be necessary.

Monitoring and supervision were stated as particularly relevant assets, including by lay counselors. This qualitative testimony is corroborated by a study by Graaff and colleagues (2020), which found that PM + delivered by non-specialist peer refugee helpers was deemed acceptable, feasible, and safe [33]. From a mentorship perspective, PIH has long prioritized a structured model of supportive supervision [34]. Similar models of clinical mentorship have likewise appeared to be promising entry points for enhancing competencies among health providers [35]. The best evidence indicates that a successful mentorship program should include rigorous mentor selection and comprehensive and consistent trainings [36]. Positive feedback from providers indicates that supportive supervision models are an integral element of task-sharing mental health interventions.

The implementation of healthcare interventions in low-income settings often presents logistical challenges for achieving optimal outcomes. In Neno District, constrained transportation and difficult terrain, significantly restricted the mobility of providers and the delivery of services. Sewell (2016) provides a conceptual framing of this narrative in which efforts to promote healthcare are dependent on the collective inputs of the municipality, for example, the establishment of roads through which ambulances can transport sick individuals [37]. The healthcare sector is not isolated from other sectors; there are underlying co-dependencies. Our experiences align with prior research, highlighting the necessity for government and local partners to prioritize infrastructure developments that allow patients, providers, and communities to access and provide essential healthcare services.

Additionally, some patients missed follow-up appointments and did not follow their regimen of care, an observation that often occurs in real-world implementation trials [38]. Part of this may have to do with the perceived weak gravity of depression, compared to physical health conditions. For example, clients living with HIV who were advised to travel to clinics twice per year tended to persist in this practice even if advised to come more often, at least as reported by providers. Chapman and colleagues (2022) discuss three barriers that may lead to missed appointments for mental healthcare: appointment disinterest, competing demands, and insufficient systems [39]. An interplay of all three is reflected in the results of the IC3D clinical trial. Some providers shared that they forgot to prescribe medication or to document medical visits, which in turn led to patients defaulting on their medication regimens. Previous research has observed similar patterns of introducing new services into existing systems and indicates a need to focus on continuity of care and patient follow-up going forward [40].

Lastly, providers proposed a series of recommendations, including expanded screening coverage for depression, along with adequate resourcing to meet this objective. In a recent study, Ng’oma and colleagues (2019) highlighted the importance of strengthening healthcare delivery at the primary care level, where many individuals initially access care [41]. In Neno District, this could include depression screening in the OPD and palliative care, among other locations. However, this would need to be balanced with ongoing resource constraints—which providers already reported in terms of transportation infrastructure and feelings of being overburdened with clinical duties. Ultimately, greater investments in human and other resources would be necessary to meet the expansive burden of disease associated with MDD.

### Limitations

We note several study limitations. First, providers may have been hesitant to share negative experiences associated with the trial due to social desirability bias when reporting information to the interviewer. To mitigate this, interviews were conducted by a researcher unaffiliated with the clinical trials supervisory structure; however, some bias may have persisted. Second, the perspectives represented in these interviews were limited to clinical officers and counselors. A broader analysis would have included data clerks and administrative staff who were also involved in aspects of service delivery or supported service delivery indirectly. Unfortunately, we did not have the resources to expand interviews to these groups. Third, as a qualitative study conducted within the context of a clinical trial, the findings may not fully reflect the challenges and facilitators that would arise in routine service delivery without trial-related resources and support. Finally, because the study was conducted in a single rural district, the findings may not be generalizable to other settings with different health system structures, resources, or patient populations.

## Conclusion

Integrating depression care into chronic care services in Neno District, Malawi, was perceived by clinical officers and psychosocial counselors as both feasible and acceptable, with clear benefits for patients’ mental, physical, and functional health. Successful implementation was supported by structured mentorship, ongoing training, standardized tools, and a holistic approach to care, while persistent challenges, such as transportation barriers, increased workload, documentation gaps, and limited resources, must be addressed to sustain and scale the model. Based on the results of this study, future research should explore strategies to improve the integrated depression model in low-resource settings, including accounting for comorbid mental health conditions, and expanding access to screening and care. Continuing to incorporate provider feedback will maximize clinical impact and improve patient outcomes. 

## Supplementary information

Below is the link to the electronic supplementary material.

Supplementary Material 1

## Data Availability

The datasets used and/or analyzed during the current study are available from the corresponding author on reasonable request.

## Abbreviations

ADT: Antidepressant Therapy
ART: Antiretroviral Therapy
EMR: Electronic Medical Record
IC3: Integrated Chronic Care Clinics
IC3D: Integrated Chronic Care Clinics, Depression
LMICs: Low- and Middle-Income Countries
MDD: Major Depressive Disorder
NDHRC: Neno District Health Research Committee
NHSRC: National Health Sciences Research Committee
OPD: Outpatient Department
PHC: Primary Health Care
PHQ-9: Patient Health Questionnaire-9
PIH/APZU: Partners in Health/Abwenzi Pa Za Umoyo
PM+: Problem Management Plus
PDC: Paediatric Development Centre
POSER: Program on Social and Economic Rights
WHO: World Health Organization
