# Supplementary material for: Provider perceptions and experiences integrating depression treatment into chronic healthcare services in Neno District, Malawi: a qualitative study
Source: BMC Health Serv Res. 2025 Dec 10;26:66. doi: 10.1186/s12913-025-13830-2 (PMC12801780; doi:10.1186/s12913-025-13830-2)
Supplement: Supplementary file 1 — Supplementary Material 1 [file 12913_2025_13830_MOESM1_ESM.docx]

**INTERVIEW PROTOCOL**

**Introduction**

1. Could you describe, from your perspective, the process of integrating depression care into the IC3?
2. What was your experience like providing group PM+ sessions to patients diagnosed with depression in Neno?

- If relevant, experience with: assessment, home visits, follow-up care

**Effectiveness**

1. Did you find group PM+ sessions effective? Explain why you think it has been effective or not, from your perspective.
2. Thinking about the practices used in group PM+ sessions, which ones did you notice to be most effective among your clients? Why do you think these were most effective?
3. Thinking about the practices used in group PM+ sessions, which ones did you notice to be least effective among your clients? Why do you think these were least effective?

**Additional Processes**

1. Can you describe your experiences with the screening and follow-up process for clients with depression?
2. For professional counselors: How has your experience been providing group PM+ compared to individual counselling sessions you have provided previously in your career?

**Challenges and Recommendations**

1. What were challenges you faced in the provision of PM+?
2. What was your experience like in terms of the supervision process (i.e. having your performance reviewed)?
   - What went well?
   - What were some challenges?
3. Would you recommend group PM+ sessions to other clients? Why/Why not?
4. Is there anything you would want to change in the way group PM+ sessions were conducted?

- If yes, explain

**Wrap-Up**

1. Do you have any final reflections or comments you would like to share?
